# Transcription Factors and microRNA-Co-Regulated Genes in Gastric Cancer Invasion in *Ex Vivo*

**DOI:** 10.1371/journal.pone.0122882

**Published:** 2015-04-10

**Authors:** Yue Shi, Jihan Wang, Zhuoyuan Xin, Zipeng Duan, Guoqing Wang, Fan Li

**Affiliations:** 1 Department of Pathogenobiology, The Key Laboratory of Zoonosis, Chinese Ministry of Education, College of Basic Medicine, Jilin University, Changchun, Jilin, China; 2 The Key Laboratory for Bionics Engineering, Ministry of Education, China, Jilin University, Changchun, China; Dana-Farber Cancer Institute, UNITED STATES

## Abstract

Aberrant miRNA expression abnormally modulates gene expression in cells and can contribute to tumorigenesis in humans. This study identified functionally relevant differentially expressed genes using the transcription factors and miRNA-co-regulated network analysis for gastric cancer. The TF-miRNA co-regulatory network was constructed based on data obtained from cDNA microarray and miRNA expression profiling of gastric cancer tissues. The network along with their co-regulated genes was analyzed using Database for Annotation, Visualization and Integrated Discovery (DAVID) and Transcriptional Regulatory Element Database (TRED). We found eighteen (17 up-regulated and 1 down-regulated) differentially expressed genes that were co-regulated by transcription factors and miRNAs. KEGG pathway analysis revealed that these genes were part of the extracellular matrix-receptor interaction and focal adhesion signaling pathways. In addition, qRT- PCR and Western blot data showed an increase in COL1A1 and decrease in NCAM1 mRNA and protein levels in gastric cancer tissues. Thus, these data provided the first evidence to illustrate that altered gene network was associated with gastric cancer invasion. Further study with a large sample size and more functional experiments is needed to confirm these data and contribute to diagnostic and treatment strategies for gastric cancer.

## Introduction

Gastric cancer is one of the most common form of malignancies in the world, contributing to a third of cancer-related deaths in men and a fifth among women [1]. Approximately, two-thirds of gastric cancer cases occur in the developing countries. In China, the incidence and mortality related to gastric cancer ranks third among other forms of malignancies [2] and it was reported that gastric cancer occurs more frequently in rural areas and with a trend of younger people being affected by it in recent years [3]. Environmental (such as *Helicobacter pylori* infection or consumption of smoked foods) and genetic factors (*E-cadherin* mutation) increases the susceptibility to gastric cancer by inducing alterations in oncogenes/tumor suppressor genes and/or epigenetic profile [4]. Alteration in these critical factors results in abnormal regulation of cell growth, apoptosis, and differentiation thus promoting carcinogenesis. Multiple gene regulatory networks co-ordinate the transformation of normal cell to a tumor cell and drive tumor progression. However, to date, the detailed understanding of the underlying multiple gene regulatory networks in pathogenesis of gastric cancer is yet to be defined. Determining the detailed molecular mechanistic network associated with gastric cancer development and progression could improve the understanding of carcinogenesis in gastric tissues, thus paving way for novel and effective strategies in the prevention, diagnosis and treatment of gastric cancer.

Gene expression in cells is controlled both at transcription and post-transcriptional levels. Transcription factors (TFs) coordinate gene transcription, while miRNAs regulates gene expression by mediating post-transcriptional events, such as mRNA degradation and protein translation [5]. Therefore, any alterations in miRNA function can result in the development of cancer in humans [6,7]. Transcription factors are proteins that bind to specific DNA sequences to control the rate of transcription of genetic information from DNA to mRNA [8,9], while miRNAs are a group of a small non-coding RNA in cells and function in RNA silencing and post-transcriptional regulation of gene expression [10,11]. The TF-miRNA gene regulatory network determines the overall gene expression profile in cells to some extent. Therefore, analysis of the TF-miRNA co-regulatory networks in gastric cancer tissues could help us to further our understanding on how TFs and miRNAs coordinate the regulation of gene expression contributing to gastric carcinogenesis [12]. In our previous study, we profiled differentially expressed genes in eighty pairs of gastric carcinoma-adjacent normal tissues using cDNA microarrays [13] and found a number of genes with altered expression, including TFs. Based on the information from Transcriptional Regulatory Element Database (TRED) [14], we built and consolidated a TF-gene regulatory network. In this study, we profiled differentially expressed miRNAs in five pairs of gastric carcinoma-adjacent normal tissues and constructed a miRNA-target regulatory network for gastric cancer by integrating the miRNA targeting gene databases, including Targetscan, miRanda, miRDB, and miRWalk [15]. We then constructed the TF-miRNA co-regulatory network using our previous data and then performed GO and KEGG pathway analyses and performed real time PCR and western blot analysis to validate these data. Thus, both of the methods and analyses could provide important clues for future studies on miRNA and TFs functions in gastric cancer.

## Materials and Methods

### Tissue samples

A total of 25 gastric carcinoma patients were recruited for this study from The First Hospital of Jilin University, Changchun, China. Gastric cancer tissues and the matching distant non-cancerous tissues were surgically resected and stored in liquid nitrogen within 10 min after the resection. Written informed consents were obtained from all the subjects and the data were analyzed anonymously. The TNM and histological classification were according to World Health Organization (WHO) criteria. This study was approved by the Ethics Committee of College of Basic Medical Sciences, Jilin University.

### Profiling of differentially expressed mRNA and microRNA in gastric cancer tissues

The differentially expressed mRNA data between gastric cancer and normal tissues was conducted from 80 patients and reported previously [13]. We used ≥ 2-fold change to profile the differentially expressed genes for this study.

In this study, differentially expressed miRNAs in 5 pairs of gastric cancer–adjacent normal tissues (see patients’ data in S2 Table) were profiled using Affymetrix miRNA microarray chips according to the manufacturer’s protocols. Briefly, total RNA from tissue samples was isolated using the Trizol (Invitrogen, Carlsbad, CA, USA) and miRNA was isolated and purified using the mirVana miRNA Isolation Kit (Ambion, Austin, TX, USA) and then subjected to Gene Chip microRNA array analysis. The data were scanned using GeneChip Scanner3000 with GeneChip Operating Software (GCOS) and analyzed.

### Construction of TF-gene, miRNA-targeting gene, and TF-miRNA co-regulatory networks

Based on the GeneChip Human Exon 1.0 ST microarray data (Affymetrix, CA, USA), we constructed the TF-gene network by integrating gene expression profiles and transcriptional regulatory element database (TRED). Regulatory interactions between microRNA and their target genes were established based on information from Targetscan, miRanda, miRDB and miRWalk database. The TF-miRNA co-regulatory networks were constructed by overlapping these two sections. Hub-genes that co-regulated by TFs and miRNAs were also identified. The networks were constructed using Cytoscape software (Institute of Systems Biology, USA, http://www.cytoscape.org).

### Functional annotations of selected genes

Online analytical tools such as Database for Annotation, Visualization and Integrated Discovery (DAVID) and Kyoto Encyclopedia of Genes and Genomes (KEGG) were applied to explore the functional pathway associated with differentially expressed genes. Significantly enriched KEGG pathways with p < 0.01 were identified and analyzed further.

### Quantitative RT-PCR (qRT-PCR)

For detect mRNA level, we utilized 5 μg total RNA samples of each sample to reversely transcribe into cDNA with the first strand cDNA Synthesis Kit (Takara, Dalian, China) and then amplified using qPCR for expression of COL1A1, and NCAM1 mRNA with SYBR Premix Ex Taq (Takara) in Applied Biosystems 7300 Fast Real-Time PCR System according to the manufacturers’ instructions. The relative expression of mRNA levels was normalized to β-actin mRNA by comparative Ct method (2^-ΔΔCt^, ΔCt = Ct _target_-Ct _β-actin_, ΔΔCt = ΔCt_tumor_-ΔCt_normal_). All primers were designed with Primer Premier 6 Software, primer sequences for amplification were listed in Table 1. Data from qRT-PCR were analyzed with GraphPad Prism Version 5.0, differences between groups were statistically evaluated by sample one-tailed Student’s t-test with p value <0.05 considered as significant.

**Table 1:** Primer sequences used for qRT-PCR amplification.

Genes	Forward primers	Reverse primers
COL1A1	5’- GAGGGCCAAGACGAAGACATC-3’	5’ CAGATCACGTCATCGCACAAC-3’
NCAM1	5’- GGCATTTACAAGTGTGTGGTTAC-3’	5’- TTGGCGCATTCTTGAACATGA-3’
β-actin	5’-CTGGAACGGTGAAGGTGACA-3’	5’-AAGGGACTTCCTGTAACAATGCA-3’

### Protein extraction and Western blotting

Tissue specimens with 1 mm^3^ in size were ground in liquid nitrogen and homogenized in a cell lysis buffer (Beyotime, Beijing, China) at 4°C for 20 min. The concentration of protein in the samples was determined using a BCA Protein Assay Kit (Bio-Rad, Hercules, CA, USA) and the proteins samples were separated by sodium dodecyl sulfate-polyacrylamide gel electrophoresis (SDS-PAGE) using 10% gel and then transferred onto a PVDF membrane (0.45 μm; Bio-Rad, Hercules, CA, USA) for 2 h. The membranes were then incubated with a rabbit anti-collagen I antibody (Novus Biologicals, Littleton, CO, USA) at a dilution of 1:1000, a mouse anti-NCAM1/CD56 antibody (Novus Biologicals) at a dilution of 1:400, or a rabbit anti-β-actin antibody (Proteintech, Chicago IL, USA) at a dilution of 1:2000 at 4°C overnight and subsequently after washing with Tris-based saline-Tween 20 (TBST), the membranes were incubated with a goat anti-rabbit IgG (Beyotime) or goat anti-mouse IgG (Proteintech) at a dilution of 1:2000 for 2 h. The protein signals were detected by autoradiography, by using enhanced chemiluminescence reagent (Beyotime, Beijing, China) followed by exposure to the radiographs. The density of protein band was quantified using a Gel Image System (Tanon, Shanghai, China) and were normalized to β-actin levels which was used as loading controls.

### Statistical analysis

LIMMA (Linear models for microarray data) based analysis was performed to identify the differentially expressed miRNAs with a cut-off-value of at least 2-fold changes (FC) with p < 0.05 and FDR < 0.05. SPSS 21.0 software (SPSS, Chicago, IL, USA) was used to perform Receiver Operating Characteristic (ROC) curve and logistic regression analysis. The sensitivity, specificity and the area under the curve (AUC) were calculated using the Med-Calc statistical software and p value < 0.05 was considered to be statistically significant. Western blotting data were analyzed by GraphPad Prism Version 5.0 (San Diego, CA, USA) and difference between tumor and normal tissues were evaluated by the one-tailed Student’s t-test and a p value < 0.05 was considered to be statistically significant.

## Results

### TF-gene regulatory network and differential expression of miRNAs in gastric cancer

**Fig 1:**
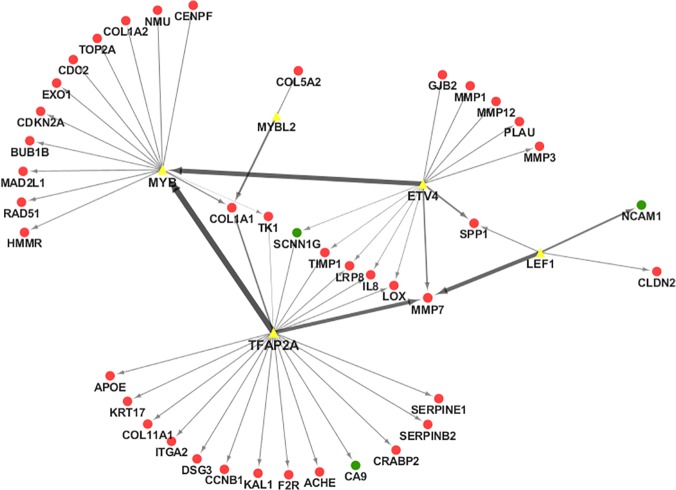
TF-gene regulatory network for gastric cancer. The network was constructed based on data from cDNA microarray analysis to identify differnetially expressed genes in gastric cancer. Red circles are up-regulated genes, while green circles are down-regulated genes and the yellow triangles represent transcription factors (TFs). The direction of the arrow is from the source to the target.

**Fig 2:**
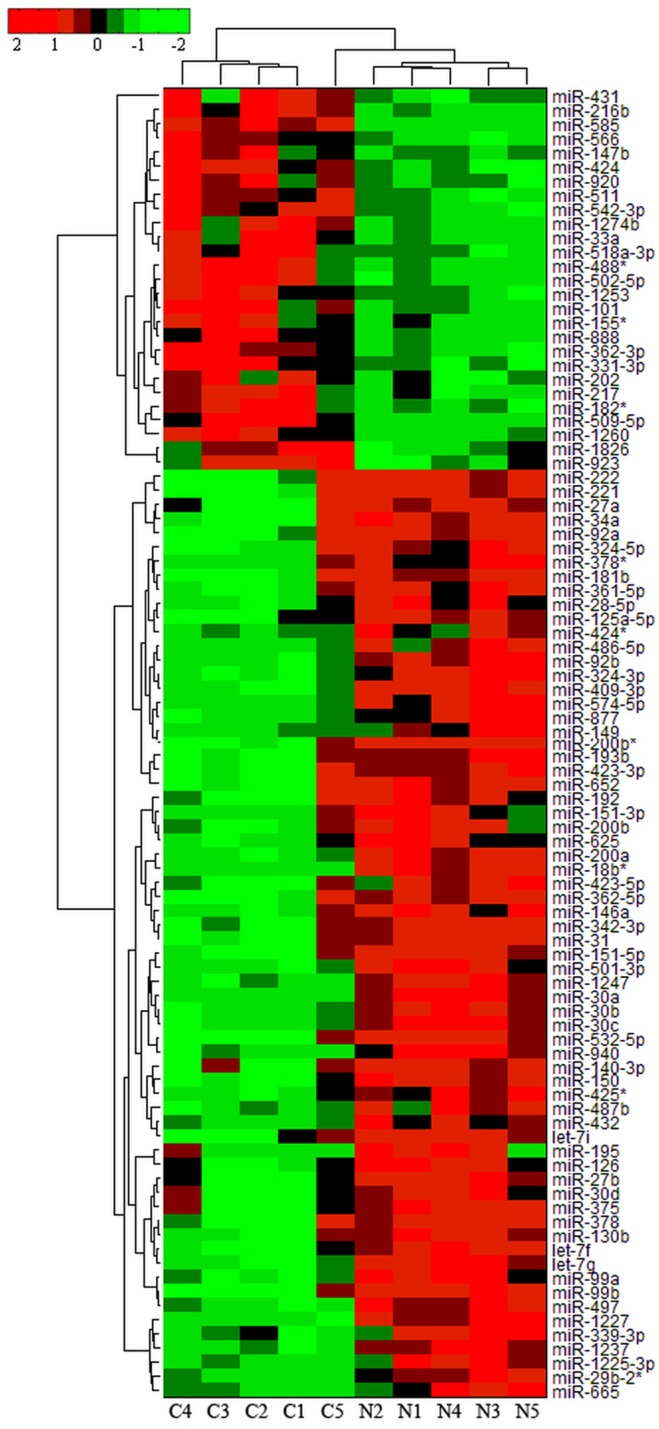
Bi-clusters analysis of differentially expressed miRNAs in gastric cancer tissues. Each row represents a miRNA and each column represents a sample. Column “C” represents cancer tissues and column “N” represent normal tissues. The heat map with red indicates up-regulated and green for down-regulated miRNAs.

TF-gene regulatory network as shown in Fig 1 was constructed based on the data obtained from a previous study [13] on differentially expressed genes (≥ 2 fold) from 80 pairs of gastric cancer tissues. Specifically, five transcription factors MYB, MYBL2, ETV4, LEF1 and TFAP2A were up-regulated and they formed the TF-gene regulatory networks with 41 genes, 38 of which were up-regulated and 3 were down-regulated in gastric cancer tissues (S1 Table). Furthermore, we profiled miRNA expression using Affymetrix microRNA arrays in five pairs of gastric cancer—corresponding normal tissues (Clinicopathological characteristics of the patients as shown in S2 Table). A total of 93 miRNAs were differentially expressed in gastric cancer tissues (p<0.05), of which 27 miRNAs were up-regulated, whereas 66 were down-regulated (Fig 2 and S3 Table). Among these differentially expressed miRNAs, several have been reported in previous studies, such as miRlet-7, miR409, miR-28-5p, miR-625, etc. [16–19]. Subsequently, Targetscan, miRanda, miRDB, and miRWalk databases were mined to predict the target genes of these differentially expressed miRNAs.

### TF-miRNA network regulating differentially expressed genes in gastric cancer

**Fig 3:**
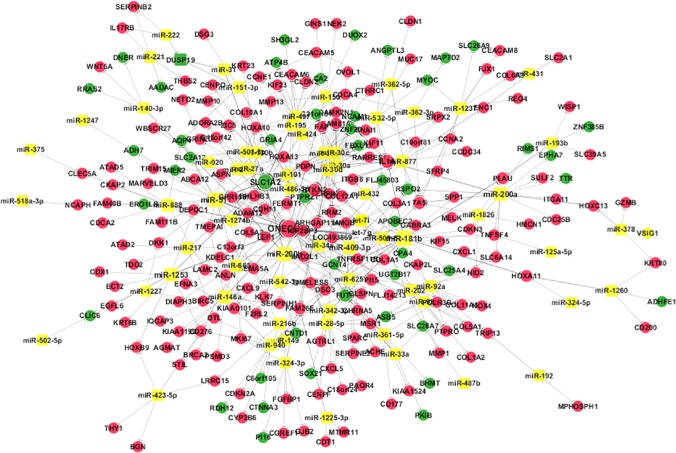
miRNA-target network for gastric cancer. Red circles are up-regulated genes, while green circles are down-regulated genes and the yellow squares represent miRNAs. Lines with “T” edges in the network represent inhibitory effect of miRNAs on target genes.

**Fig 4:**
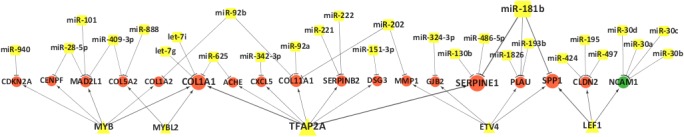
Hub-genes in the TF-miRNA co-regulatory network. Circles in the central line are genes that co-regulated by TFs and miRNAs, with red for up-regulated genes and green for down-regulated genes. Yellow squares represent miRNAs and yellow triangles represent TFs.

Based on the datasets from cDNA and miRNA microarray as mentioned earlier, we constructed an aberrant TF-miRNA network that regulated the expression of genes in gastric cancer (Fig 3 and S4 Table). Particularly, these aberrant TF-miRNA networks regulated expression of 18 genes (*COL1A1*, *COL1A2*, *COL5A2*, *COL11A1*, *DSG3*, *ACHE*, *SERPINE1*, *SERPINB2*, *CXCL5*, *MMP1*, *PLAU*, *SPP1*, *GJB2*, *CLDN2*, *CDKN2A*, *CENPF*, *MAD2L1*, and *NCAM1*), most of which (17 out of 18) were up-regulated in gastric cancer tissues (Fig 4).

**Fig 5:**
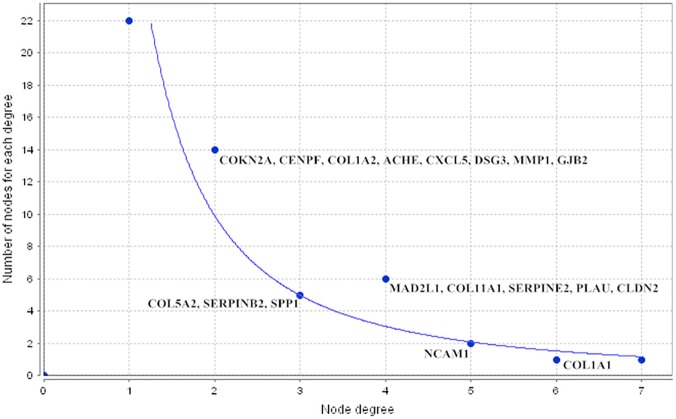
Node degree distribution of miRNA-TF co-regulatory network. These 18 target genes were co-regulated by both of TFs and miRNAs with their corresponding node degree. The X axis shows the degree of a node, while the Y axis shows the number of node for each degree in the network.

Functional analysis of these 18 hub-genes using DAVID (the Database for Annotation, Visualization and Integrated Discovery)[20] showed that there were two significantly enriched KEGG pathways, the ECM-receptor interaction pathway and focal adhesion pathway. Five genes (*COL1A1*, *COL1A2*, *COL5A2*, *COL11A1*, and *SPP1*) were most significantly altered and were all involved in the ECM-receptor interaction and focal adhesion pathway (Table 2). Analysis of the co-regulatory network showed that these 18 hub-genes had a different node degree distribution, while *COL1A1* and *NCAM1* showed the highest degree distribution (Fig 5).

**Table 2:** Significant KEGG pathways of the hub-genes regulated by both TFs and miRNAs.

Functional Annotation	*p*-value	Benjamini	Genes
hsa04512: ECM-receptor interaction	4.42E-05	0.001017	*COL1A2*, *COL1A1*, *COL5A2*, *COL11A1*, *SPP1*
hsa04510: Focal adhesion	0.001279	0.014609	*COL1A2*, *COL1A1*, *COL5A2*, *COL11A1*, *SPP1*

### Association of *COL1A1* and *NCAM1* expressions with clinicopathological status

**Fig 6:**
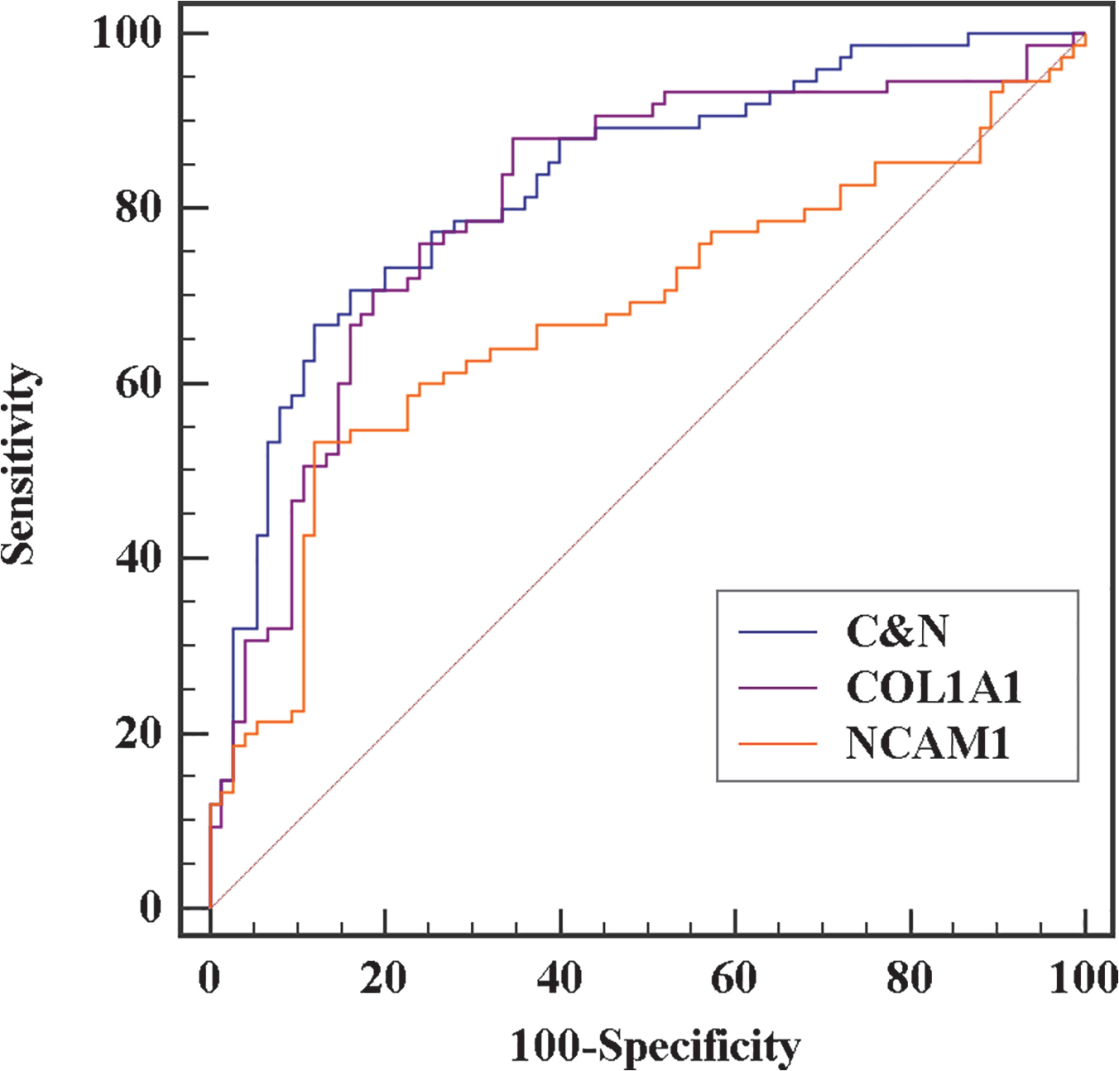
ROC curve of genes as discriminators between gastric cancer and normal tissues. C&N indicates the combination of *COL1A1* and *NCAM1*.

We assumed that genes with higher degree distributions could play a major role in the regulatory network. Thus, we associated expression of these genes with clinicopathological characteristics of gastric cancer patients. The receiver operating characteristic (ROC) curve analysis showed that expression of *COL1A1* and *NCAM1* could be potential discriminators between cancer and corresponding normal tissues with AUC (area under curve) = 0.806 for *COL1A1* and 0.677 for *NCAM1*. The combination of *COL1A1* and *NCAM1* expression provided a better differentiation condition with AUC = 0.829, sensitivity = 70.7% and specificity = 84.0% than that of individual *COL1A1* or *NCAM1* expression (Fig 6 and Table 3).

**Table 3:** ROC analysis using the Med-Calc software.

Genes	Sensitivity	Specificity	AUC	SE	*p*-value
*COL1A1*	88.0	65.3	0.806	0.0369	<0.0001
*NCAM1*	53.3	88.0	0.677	0.0450	0.0001
C&N	70.7	84.0	0.829	0.0335	<0.0001

AUC, Area under the ROC Curve; SE, Standard error; C&N, combination of *COL1A1* and *NCAM1*.

**Fig 7:**
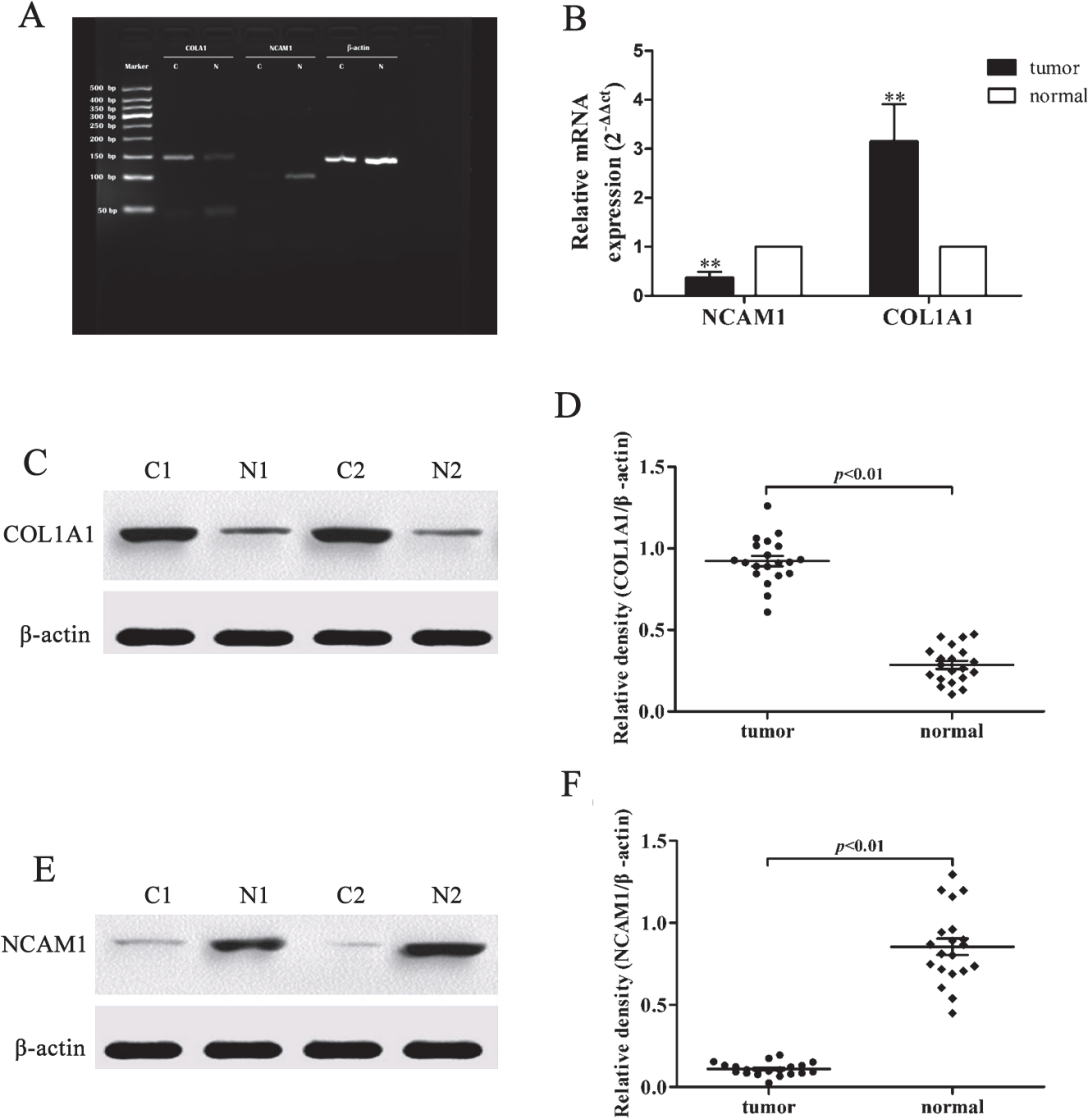
Validation of COL1A1 and NCAM1 expression in 20 pairs of gastric cancer and normal tissues. A and B, Detection of COL1A1 and NCAM1 mRNA expression in gastric cancer vs. normal tissues using PCR and qRT-PCR. Levels of COL1A1, NCAM1 mRNA were 3.10 ± 1.08 folds up-regulation and 0.37 ± 0.02 folds down-regulation in tumor tissues, respectively compared to those of the normal ones. *p<0.01. C and D, Western blot analysis of COL1A1 protein. Tumor tissues expressed higher level of COL1A1 protein compared to the normal ones (p<0.01). E and F, Western blot analysis of NCAM1 protein. Tumor tissues expressed lower level of NCAM1 protein compared to the normal ones (p<0.01). N, normal tissues; C, cancer tissues.

**Fig 8:**
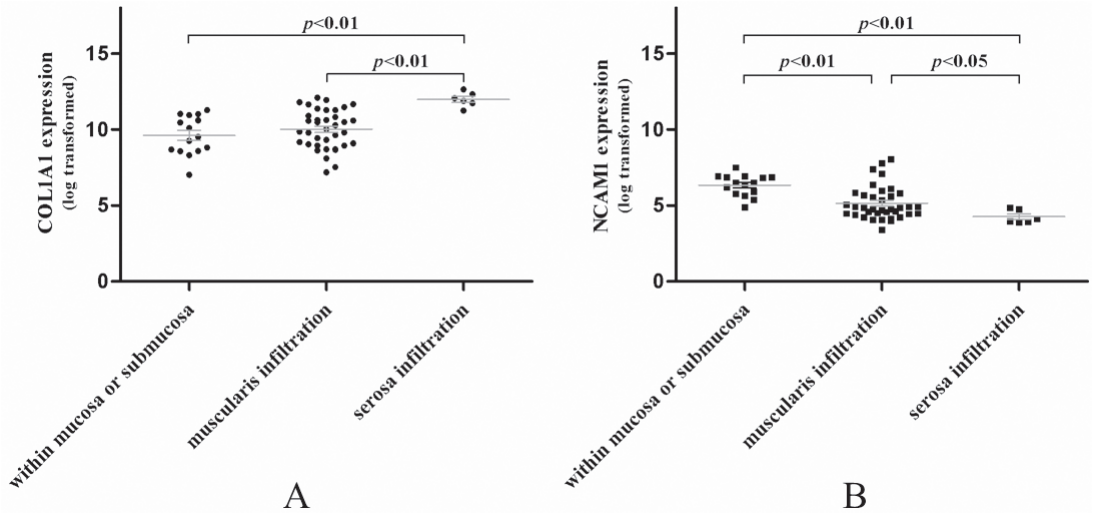
Expression of COL1A1 and NCAM1 associated with gastric cancer invasion depth.

Furthermore, we validated the microarray data using qRT-PCR and Western blot in other 20 pairs of gastric cancer and adjacent normal tissues (patients’ information listed in S2 Table). The COL1A1 and NCAM1 mRNA expression showed 3.10 ± 1.08 fold up-regulation and 0.37 ± 0.02 fold down-regulation in tumor tissues vs. normal ones (p < 0.01), while Western blot data showed a clear difference between the relative protein density of COL1A1 in cancer tissues (0.92 ± 0.02) vs. adjacent normal tissues (0.29 ± 0.01; p < 0.01), while expression of NCAM1 in cancer tissues (0.11 ± 0.002) vs. normal ones (0.85 ± 0.05) (p< 0.01, Fig 7). Thus, the up-regulation of COL1A1 and down-regulation of NCAM1 expression could not only distinguish between cancer and normal tissues, but also divide the cancer patients into different tumor stages. The level of COL1A1 expression was higher in the muscular and serosa invaded tumors, while NCAM1 expression tended to be negatively associated with tumor invasion (Fig 8).

## Discussion

In the current study, data from the cDNA and miRNA microarray was used to construct the transcription factors-miRNA co-regulatory network in gastric cancer and identified 18 hub-genes that were regulated by both transcription factors and miRNAs. These genes belong to extracellular matrix-receptor interaction and focal adhesion signaling pathways. In addition, the expression of *COL1A1* and *NCAM1* was confirmed in gastric cancer tissues and were associated with gastric cancer invasion; however, it remains unknown which miRNA(s) regulate their expression in gastric cancer.

Transcription factors MYB, MYBL2, ETV4, LEF1,TFAP2A were up-regulated in gastric cancer tissues. Indeed, the MYB family proteins are widely distributed in eukaryotic organisms and expresison of MYB-transcription factor is critical for tumor growth and mammary carcinogenesis [21] [22], while MYBL2 (B-MYB) is an oncogenic transcription factor involved in cell cycle, G2/M progression [23]. As a member of oncogenic *ETS* genes, ETV4 protien has been reported to promote cancer metastasis in mouse models [24] and is associated with poor prognosis in gastric adenocarcinoma [25]. The TCF/LEF family is a small family of DNA-binding factors and LEF1 acts mainly as an activator with a role in inhibition of cell apoptosis [26]. TFAP2A is a transcription factor that mainly regulates cell growth and differentiation. In nasopharyngeal carcinoma, TFAP2A regulated tumor cell growth and survival through the HIF-1α-mediated VEGF/PEDF signaling pathway, suggesting that TFAP2A could be a potential biomarker for nasopharyngeal carcinoma treatment [27]. Furthermore, in the miRNA-TF co-regulatory network, we identified 18 hub-genes that were regulated by both TFs and miRNAs. Functional analysis of these 18 genes highlighted two significant KEGG pathways, the extracellular matrix (ECM)-receptor interaction pathway and focal adhesion pathway. Recent studies demonstrated that ECM-receptors (Integrins) mediated signaling are a major group of signals contributing to cell survival and provides a survival advantage to various types of cancer cells [28]. ECM can also regulate cell proliferation, differentiation, death and carcinogenesis [29]. As the structural links between ECM and actin cytoskeleton, focal adhesions serves as sites for signal transduction from the ECM to intracellular compartment [30]. Our current data showed that the five genes (*COL1A1*, *COL1A2*, *COL5A2*, *COL11A1*, and *SPP1*) co-regulated by both TFs and miRNAs participated in ECM-receptor interaction and focal adhesion pathways. Previous study showed overexpression of *SPP1* (secreted phosphoprotein 1) in gastric cancers and its association with cancer progression [31]. The genes *COL1A1*, *COL1A2*, *COL5A2*, and *COL11A1* belong to the collagen protein family, essential structural components of ECM. Up-regulation of collagens is crucial to promote tumor growth as collagen catabolized by matrix metalloproteinases (MMPs) reveals the hidden binding sites that further promote angiogenesis and tumor invasion. A previous study showed that expression of COL1A1 and COL1A2 was elevated in malignant colorectal endothelium cells [32], suggesting that these two proteins play a role in angiogenesis and formation of desmoplasia during colorectal cancer development [33]. Moreover, expression of *COL5A2* and *COL11A1* was associated with colorectal carcinogenesis [34] showing that *COL5A2* was co-expressed with *COL11A1* in colorectal tumor samples, but not in normal colon epithelia; however, it remains unknown which miRNA(s) regulate their expression in gastric cancer. Depth of cancer invasion is an important factor in the prediction of survival and treatment planning. Collagen is one of the important components in the tumor microenvironment, the experimental results of subtractive hybridization and microarray indicated a variety of collagen genes that were abnormally expressed in tumor tissues, such as COL1A1 encoding type 1 collagen [35]. *COL1A1* has been identified to associate with gastric cancer invasion and metastasis [33]. Our current data confirmed that expression of COL1A1 was significantly elevated in gastric cancer tissues and is associated with tumor progression. Moreover, our current study also showed that expression of NCAM1 protein was negatively associated with gastric cancer invasion. NCAM is a multifunctional membrane protein involved in cell differentiation, migration, neural synapse growth, and special patterns of synaptic connections. A previous study reported that NCAM1 expression was associated with the invasive growth of glioma [36]. After inoculating the transfected stellate glioma cells into the brain of rats, Edvardsen *et al*., reported that the invasiveness of tumor cells reduced, indicating that level of NCAM1 expression was negatively associated with tumor invasiveness [37]. Although loss of NCAM1 expression in gastric cancer has not been reported before, our current data on the inverse association with gastric cancer invasion is consistent with previous studies of gliomas [37]. Further studies are required to confirm the expression status of COL1A1 and NCAM1 proteins as potential biomarkers for early diagnosis and prediction of gastric cancer progression.

Construction of the TF-miRNA co-regulatory network is a useful tool in the identification of critical regulators and their target genes in human cancers. However, our current study is just a proof-of-principle effort and future studies with a larger sample size are necessary to confirm the current findings. It should be followed by mechanistic studies to further the understanding about the role of the key molecules and gene pathways in gastric cancer.

## Supporting Information

S1 Table Summary of the regulatory interactions of TF-gene network. (XLSX)

S2 Table Patients characteristics (25 pairs of gastric cancer and adjacent normal tissues for the miRNA microarray (n = 5) and Western blot (n = 20) analysis and RT-qPCR (n = 20) analysis). (DOC)

S3 Table Summary of 93 differentially expressed miRNAs in gastric cancer tissues vs. the distant normal tissues. Gene expression levels in gastric cancer tissues vs. the distant normal tissues were at least 2-fold different with a p-value <0.05.
(XLS)

S4 Table The interactions of miRNAs and their regulated genes in TF-gene regulatory network. All regulation was derived from transcriptional regulatory element database (TRED).
(XLSX)
