# Supplementary material for: Transcription Factors and microRNA-Co-Regulated Genes in Gastric Cancer Invasion in Ex Vivo
Source: PLoS One. 2015 Apr 10;10(4):e0122882. doi: 10.1371/journal.pone.0122882 (PMC4393113; doi:10.1371/journal.pone.0122882)
Supplement: S2 Table — (DOC) [file pone.0122882.s002.doc]

Table S2. Clinicopathological characteristics of the study samples

| Case | Participate | Clinical parameters | | | |
| --- | --- | --- | --- | --- | --- |
|  |  | ***Gender*** | ***Age*** | ***Differentiation status*** | ***Clinical stage*** |
| 1 | microarray | Female | 46 | Poorly differentiated | IV |
| 2 | microarray | Male | 55 | Moderately differentiated | III |
| 3 | microarray | Female | 49 | Moderately differentiated | III |
| 4 | microarray | Female | 69 | Highly differentiated | II |
| 5 | microarray | Female | 63 | Highly differentiated | I |
| 6 | Western blot and RT-qPCR | Male | 47 | Poorly differentiated | III |
| 7 | Western blot and RT-qPCR | Male | 71 | Moderately differentiated | III |
| 8 | Western blot and RT-qPCR | Female | 65 | Poorly differentiated | IV |
| 9 | Western blot and RT-qPCR | Male | 66 | Highly differentiated | II |
| 10 | Western blot and RT-qPCR | Female | 57 | Moderately differentiated | III |
| 11 | Western blot and RT-qPCR | Female | 54 | Highly differentiated | II |
| 12 | Western blot and RT-qPCR | Male | 60 | Poorly differentiated | IV |
| 13 | Western blot and RT-qPCR | Female | 63 | Moderately differentiated | III |
| 14 | Western blot and RT-qPCR | Male | 72 | Moderately differentiated | III |
| 15  16  17  18  19  20  21  22  23  24  25 | Western blot and RT-qPCR Western blot and RT-qPCR  Western blot and RT-qPCR Western blot and RT-qPCR  Western blot and RT-qPCR Western blot and RT-qPCR Western blot and RT-qPCR  Western blot and RT-qPCR Western blot and RT-qPCR Western blot and RT-qPCR Western blot and RT-qPCR | Male  Male  Male  Male  Female  Male  Male  Male  Female  Female  Male | 50  42  60  47  62  62  69  57  41  70  70 | Highly differentiated  Poorly differentiated  Poorly differentiated  Poorly differentiated  Moderately differentiated  Poorly differentiated  Highly differentiated  Moderately differentiated  Highly differentiated  Moderately differentiated  Moderately differentiated | II  II  III  III  III  III  III  II  III  II  III |
